# Budget Impact Analysis of the Use of Specific Biomarkers GFAP and UCH-L1 in the Management of Mild Traumatic Brain Injury in Spain

**DOI:** 10.3390/jcm14124095

**Published:** 2025-06-10

**Authors:** Francisco Moya Torrecilla, Gemma Álvarez-Corral, Eva Gutiérrez Pérez, Daniel Morell-Garcia, Juan Ortega Pérez, Beatriz Miriam Rodríguez, Leticia Sánchez Martín, Francisco Temboury Ruiz

**Affiliations:** 1 FML Emergency Medicine Department, Quirónsalud Málaga Hospital, 29004 Málaga, Spain; 2 Sport and Med Clinic, 29640 Málaga, Spain; 3 Clinical Laboratory Department, Hospital Universitario Virgen de las Nieves, 18014 Granada, Spain; gemma_ac@yahoo.es; 4 Instituto de Investigación Biosanitaria ibs.GRANADA, 18012 Granada, Spain; 5 Emergency Department, Hospital Universitario Virgen de las Nieves, 18014 Granada, Spain; woselina@hotmail.com; 6 Clinical Laboratory Department and Health Research Institute of the Balearic Islands (IdISBa), Hospital Universitario Son Espases, 07120 Mallorca, Spain; dr.morell.uab@gmail.com; 7 Emergency Department, Hospital Universitario Son Espases, 07120 Mallorca, Spain; juan.ortega@ssib.es; 8 Radiology Department, Hospital Universitario Son Espases, 07120 Mallorca, Spain; beatrizmiriam.rodriguezchikri@ssib.es; 9 Science & Innovation Link Office (SILO), C. de Claudio Coello 52, Planta 1, 28001 Madrid, Spain; leticia.sanchez@silocompany.com; 10 Emergency Department, Hospital Universitario Virgen de la Victoria, 29010 Málaga, Spain; pacotemboury@gmail.com

**Keywords:** mild traumatic brain injury, intracranial injury, biomarkers, budget impact analysis, computed tomography, emergency department

## Abstract

**Objective:** To evaluate the economic impact associated with the use of specific brain biomarkers glial fibrillary acid protein (GFAP) and ubiquitin C-terminal hydrolase L1 (UCH-L1) in adult patients with suspected mild traumatic brain injury (TBI) in a standard Spanish hospital setting. **Methods:** We used a budget impact analysis (BIA) to compare the cost of standard of care using head computed tomography (CT) to evaluate intracranial injury with a scenario incorporating specific biomarkers GFAP and UCH-L1 in an estimated population of 3500 adult patients attending the hospital emergency department with a score of 13 to 15 on the Glasgow Coma Scale (GCS). The probabilities associated with clinical procedures were obtained from a multidisciplinary group of experts from Spanish hospitals and supplemented with data from the literature. Costs were estimated using hospital tariffs from the Spanish autonomous communities and other official sources. **Results:** The incorporation of specific biomarkers GFAP and UCH-L1 in the management of mild TBI could generate an estimated annual savings of EUR 696,634 in a standard Spanish hospital, mainly due to reduced CT use. The average savings per patient would be EUR 199.04, and the care time would be reduced by 111 min. Sensitivity analysis, with variations of ±20% in the parameters, confirms these savings. **Conclusions:** This study suggests that the use of specific biomarkers GFAP and UCH-L1 in the management of mild TBI patients in Spain could reduce the average cost per patient, generating significant savings for hospitals. Future studies that incorporate data from clinical records will help validate these results.

## 1. Introduction

Traumatic brain injury (TBI) is the leading cause of death and disability worldwide among all traumatic injuries and is costly for both the healthcare system and society [1,2,3]. Some studies estimate that, globally, there are between 50 and 60 million new cases of TBI every year, of which more than 90% are mild [3,4].

Computed tomography (CT) scan is currently the standard diagnostic tool in the assessment of intracranial injury in patients with TBI and the identification of individuals who need immediate surgery [5,6]. There is a general consensus on performing a head CT in patients with moderate or severe TBI, but there is no agreement on the use of this procedure in patients with mild TBI (score on the Glasgow Coma Scale—GCS—between 13 and 15), given the low prevalence of intracranial abnormalities detected by CT and the low associated mortality in this population [7]. The need to identify patients with mild TBI at high risk of acute intracranial injury (AII), coupled with the lack of objective diagnostic tools to determine the neurocognitive status of patients, has led to an exponential increase in head CT requests in hospital emergency departments (ED) [7]. This situation results in not only increased radiation exposure in patients and saturated EDs but also has a significant economic impact on health systems [7,8,9].

There have been recent advances in the study of biomarkers in blood that improve the clinical characterization of patients with possible brain damage. Among these, the rapid serum/plasma test for specific biomarkers glial fibrillary acid protein (GFAP) and ubiquitin C-terminal hydrolase L1 (UCH-L1) for mild TBI represents a paradigm shift in the evaluation of the lesion [7]. The inclusion of this test in the clinical evaluation of adult patients with GCS 13–15 in the first 12 h after the TBI guides the need for scans, reserving the use of CT for necessary cases [7,10].

The Spanish Society of Emergency Medicine (SEMES) recently collaborated with another five scientific societies in the publication of a set of guidelines, based on an updated, mutually agreed algorithm to standardize the management of patients with mild TBI and incorporate the use of specific biomarkers GFAP and UCH-L1 in routine clinical practice [7]. This approach offers a more detailed risk stratification, aimed at defining best patient management and optimizing the use of resources. Nevertheless, economic evidence on the incorporation of specific biomarkers GFAP and UCH-L1 in the Spanish setting is limited.

The objective of this study was to evaluate the economic impact associated with the use of specific biomarkers GFAP and UCH-L1 in adult patients with GCS 13–15 admitted to an ED, in a standard Spanish hospital setting. The secondary objective was to analyze the impact on management times for the different processes involved.

## 2. Materials and Methods

A budget impact analysis (BIA) model was developed to evaluate the economic impact associated with the use of specific biomarkers GFAP and UCH-L1 in patients with suspected mild TBI admitted to the ED. The direct costs, updated to 2023, and time dedicated to the management of these patients were quantified. The model was developed following specific methodological guidelines and recommendations for studies of this type [11,12,13].

### 2.1. Study Population, Perspective, and Timelines

This study included patients aged 18 years or older with suspected mild TBI, defined by a GCS score of 13–15, who attended the ED. The BIA model was developed from the perspective of a Spanish hospital. The timeline of this study was 1 year, and the population with suspected mild TBI seen in a standard Spanish hospital was estimated to be approximately 3500 patients per year.

### 2.2. Structure of the Model and Comparators

**Figure 1:**
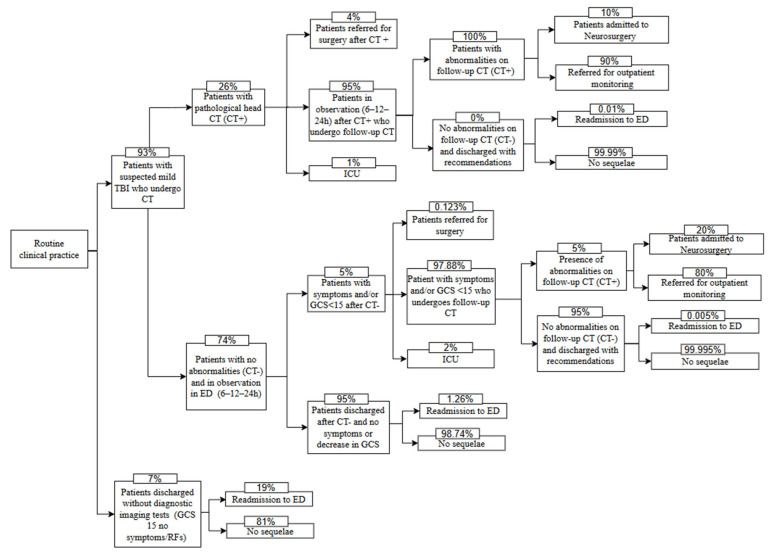
Scenario without the use of specific biomarkers GFAP and UCH-L1. GCS, Glasgow Coma Scale; RFs, risk factors; ED, emergency department; CT, computed tomography; ICU, intensive care unit.

**Figure 2:**
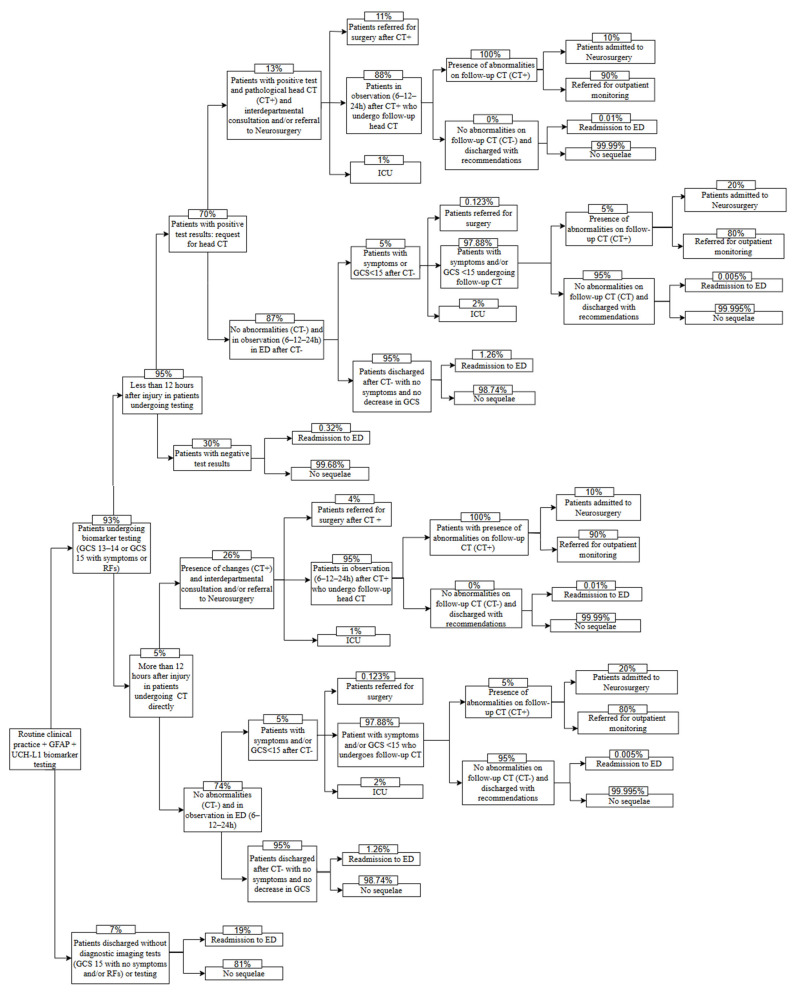
Scenario using specific biomarkers GFAP and UCH-L1. GFAP, glial fibrillary acid protein; UCH-L1, ubiquitin C-terminal hydrolase L1; GCS, Glasgow Coma Scale; RFs, risk factors; ED, emergency department; CT, computed tomography; ICU, intensive care unit.

The BIA model is based on a decision tree developed in Microsoft Excel. It was constructed from the SEMES recommendations on the use of the specific biomarkers GFAP and UCH-L1, published in December 2023, aimed at standardizing the management of patients with mild TBI in Spain [7]. The decision tree compared 2 scenarios: standard of care, which consists of performing a head CT to evaluate possible AII (Figure 1), and the updated scenario, which incorporates the use of specific biomarkers GFAP and UCH-L1 in patients with suspected mild TBI who attend the ED within 12 h of injury (Figure 2) [10,14]. The proposed model compares the average cost per patient for each of the 2 scenarios.

### 2.3. Identification, Quantification, and Evaluation of Clinical Inputs

The probabilities associated with the clinical procedures included in the model (Table 1) were based on estimates provided by a group of experts from various specialties (emergency medicine, radiology, clinical laboratory, and clinical biochemistry), based on their practical experience with specific biomarkers GFAP and UCH-L1 in Spanish hospitals. The values in Table 1 indicate the estimated probability that each clinical input or event occurs within the modeled patient population. In cases where no information was available, a literature review was used to complete the data.

### 2.4. Cost Estimation

To estimate the costs of the procedures included (Table 2), we examined data from the official tariffs of the most representative autonomous communities and official sources, such as the Minimum Basic Data Set (MBDS) [16], which collects clinical and service usage information, and the Spanish Network of Hospital Costs (RECH) [17]. All costs are expressed in euros updated to the year 2023. The economic modeling of the cost of the specific biomarkers GFAP and UCH-L1 was calculated based on the experience of their use in 1 hospital over a period of 2 years. During this time, 2 annual calibrations were performed due to a change in lot number; 1 control level was run daily, and between 15 and 20 measurements were performed per day. Under these specific conditions of use and efficiency, the reagent efficiency is 94%.

### 2.5. Management Time Estimation

To estimate the time required to manage these patients in both scenarios, we used the times reported by the experts for the different stages of the care process, based on their own experience in clinical practice in Spain (Table 3).

### 2.6. Sensitivity Analysis

A univariate sensitivity analysis was performed to address the uncertainty associated with both the structure and the parameters of the model and to evaluate the robustness of the results. In this analysis, the values of the parameters were modified within a range of ±20%, in order to identify the variables that have the greatest impact on the savings generated by the use of specific biomarkers GFAP and UCH-L1, compared to the standard management of patients with mild TBI attending the ED.

## 3. Results

### 3.1. Budgetary Impact

The use of specific biomarkers GFAP and UCH-L1 results in a reduction in the number of CT scans performed in patients with suspected mild TBI attending the ED (in routine clinical practice, 93% of these patients would undergo a CT scan, whereas, with the use of specific biomarkers GFAP and UCH-L1, only 66.5% would undergo the procedure). Taking the estimated costs into account, Table 4 shows the average cost for each procedure included in the decision tree.

The estimated annual cost for managing patients with mild TBI in a standard hospital setting in Spain has been estimated at EUR 3,145,712 for the current scenario in which CT is the standard tool for diagnosis. This cost falls to EUR 2,449,078 when specific biomarkers GFAP and UCH-L1 are incorporated. This translates to an annual savings of EUR 696,634 for the estimated 3500 patients seen in EDs for mild TBI. The average cost per patient is EUR 899 for the standard of care with CT, compared to EUR 700 for specific biomarkers GFAP and UCH-L1, yielding a savings of EUR 199.04 per patient.

### 3.2. Impact on Management Times

Taking into account the calculated times and the probabilities included in the decision tree, the estimated management time per TBI patient in Spain is 577 min (approximately 9.5 h) in a routine clinical practice setting. When specific biomarkers GFAP and UCH-L1 are incorporated into the process, the time is reduced to 466 min (approximately 7.5 h). This translates to an average savings of 111 min per patient or 1 h and 51 min.

### 3.3. Sensitivity Analysis Results

**Figure 3:**
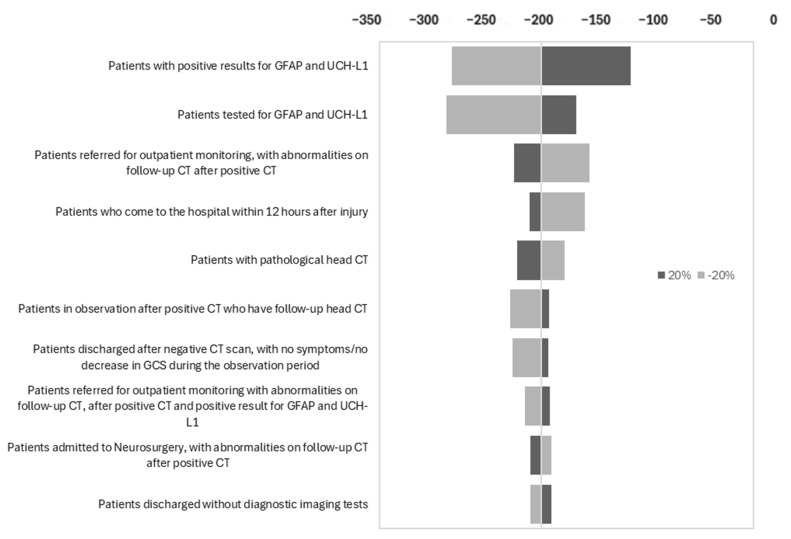
Univariate sensitivity analysis. Impact of variables on cost difference.

The parameters that most influence the savings generated by the addition of specific biomarkers GFAP and UCH-L1 are shown at the top of Figure 3. The variables with the greatest impact were the percentage of patients with positive results for specific biomarkers GFAP and UCH-L1 and the percentage of patients in whom these were measured. These variables proved to be the most sensitive for determining the savings generated by the use of specific biomarkers GFAP and UCH-L1 since small variations in these parameters led to significant changes in the cost difference between the two strategies (with and without biomarkers). Despite these variations, the results of the sensitivity analysis show that the use of specific biomarkers GFAP and UCH-L1 consistently generates economic savings compared to management based on the use of CT.

## 4. Discussion

The results suggest that testing for specific biomarkers GFAP and UCH-L1 during the evaluation of adult patients with GCS 13–15 in the first 12 h after TBI could generate a significant reduction in costs, with annual savings per patient of EUR 199.04. These savings may be explained by the reduction in the number of CTs performed, along with other factors, such as a reduced need for observation in the ED.

Other studies have also recently evaluated the use of biomarkers, although their results vary depending on the context and the methodologies used. In the United States, Ruan et al. (2009) [15] evaluated the economic impact of determining S-100B before CT in the management of adult patients with mild TBI. However, the authors do not report a reduction in hospital costs because the low specificity of this test in detecting abnormal intracranial lesions fails to generate significant savings (USD 281 versus USD 160). Still, sensitivity analyses showed that the incorporation of this biomarker could result in savings if the percentage of patients undergoing CT is greater than 78%. This limited economic impact could be consistent with other diagnostic limitations of S-100B reported, particularly, its short half-life (about 30–90 min) and the variation in blood concentrations according to skin pigmentation. In contrast, specific biomarkers GFAP and UCH-L1 offer more consistent performance across diverse populations and are recommended for use within 12 h after injury [18,19,20].

In addition, results from the BRAINI study confirmed that the cutoff values for specific biomarkers GFAP and UCH-L1 could be used to rule out the presence of an intracranial injury, outperforming the S-100B assay if blood sampling occurred after 3 h after trauma [21].

In line with these findings, Zimmer et al. (2023) [22] evaluated the combined use of specific biomarkers GFAP and UCH-L1 versus CT in a French setting. Specifically, patients evaluated with specific biomarkers GFAP and UCH-L1 received fewer CTs (770.88 versus 1096.30 per 1000 patients).

Most recently, a meta-analysis conducted by Puravet et al. (2025) [18] assessed the prognostic value of the association of specific biomarkers GFAP and UCH-L1 in predicting intracranial lesions in adults after mild TBI. The study found that a combined measurement of GFAP and UCH-L1 allows for the exclusion of intracranial injury with 100% sensitivity and negative predictive value, potentially reducing the number of unnecessary CT scans by 31%.

These findings support evidence that the use of biomarkers can optimize the management of mild TBI, although differences in care pathways and clinical practice between different healthcare systems can significantly influence the results obtained. The organizational structure, clinical protocols, and guidelines vary considerably depending on the healthcare context of each country, underlining the importance of adapting economic analyses to the specific national framework.

This study, based on the consensus published by SEMES and experts from five other scientific societies on the management of mild TBI [7], is the first to evaluate the economic impact of specific biomarkers GFAP and UCH-L1 in the Spanish setting. This is a significant and fundamental advance in optimizing decision making by implementing efficient tool adoption in a setting marked by limited resources and increasing pressure on the healthcare system.

It is important to note that the adjustment made for the cost of the test, following the recommendations of the national experts, reflects an optimal scenario that might not necessarily be the case in laboratories with less specialized protocols. Factors such as frequency of use, number of controls required, and staff experience directly affect the real achievable test efficiency in the laboratory setting. These results should, therefore, be interpreted as the upper limit of efficiency in a highly optimized setting.

Nevertheless, sensitivity analysis shows that, despite variations in the most sensitive parameters, the savings generated by the use of this test remain consistent. These observations support the robustness of the model since the use of specific biomarkers GFAP and UCH-L1 remains a more economical strategy than standard management with CT.

### Study Limitations

This study has some limitations. The data were not obtained from a retrospective analysis based on clinical practice data. The incorporation of real clinical records in future studies would help validate the data used in this study, thus providing more accurate results, while also testing the hypotheses and robustness in real clinical practice. Economic savings could be greater if indirect costs, such as travel, waiting times, and losses in labor productivity, that were not included in this analysis, were taken into account. This clearly highlights the need for future studies that quantify these dimensions.

Another factor to bear in mind, as pointed out by some researchers (Ladang A., et al. 2024) [23], is that a cut-off point adjusted to different age groups could influence the results of specific biomarkers GFAP and UCH-L1. However, additional studies are required to confirm this hypothesis. For now, the results of this study confirm that the incorporation of specific biomarkers GFAP and UCH-L1 testing could decrease the use of CT, resulting in a significant reduction in ED costs and management times in Spain.

## 5. Conclusions

This study suggests that the use of specific biomarkers GFAP and UCH-L1 in the management of patients with suspected mild TBI, according to the consensus of SEMES and experts from five other scientific societies, could not only generate significant direct economic savings but also optimize care times in a high-pressure care setting. Although this analysis has focused on the hospital setting, further studies should be performed to estimate the potential savings for the Spanish national health system, given the high prevalence of TBI.

## Figures and Tables

**Table 1:** Clinical inputs used in budget impact analysis for patients with mild TBI.

Description	Value (%)	Source
Patients discharged without diagnostic imaging tests *	7.00	RCP experience
Patients readmitted to the ED after discharge without any type of test	19.00	RCP experience
Patients with pathological head CT	26.00	RCP experience
Patients discharged after negative CT scan, no symptoms/no decrease in GCS during the observation period	95.00	RCP experience
Patients referred for surgery after negative CT but with symptoms/decrease in GCS	0.123	Ruan et al. (2009) [15]
Patients admitted to the ICU after negative CT with symptoms/decrease in GCS	2.00	RCP experience
Symptomatic patients/GCS < 15 who undergo follow-up CT after negative CT	97.88	- **
Patients with no abnormalities on follow-up CT and discharged after negative CT	95.00	RCP experience
Patients readmitted to the ED after negative CT	1.26	Ruan et al. (2009) [15]
Patients admitted to neurosurgery, with abnormalities on follow-up CT, after negative CT	20.00	RCP experience
Patients referred for outpatient monitoring, with abnormalities on follow-up CT, after negative CT	80.00	RCP experience
Patients readmitted to the ED after negative follow-up CT and negative CT	0.005	Ruan et al. (2009) [15]
Patients referred for surgery after positive CT	4.00	RCP experience
Patients admitted to ICU after positive CT	1.00	RCP experience
Patients in observation after positive CT who have follow-up head CT	95.00	- **
Patients with abnormalities on follow-up CT, after positive CT	100.00	RCP experience
Patients admitted to Neurosurgery, with abnormalities on follow-up CT, after positive CT	10.00	RCP experience
Patients referred for outpatient monitoring, with abnormalities on follow-up CT, after positive CT	90.00	RCP experience
Patients readmitted to the ED after positive CT and negative follow-up CT	0.01	Ruan et al. (2009) [15]
Patients tested for specific biomarkers GFAP and UCH-L1	93.00	RCP experience
Patients who come to the hospital within 12 h after sustaining injury	95.00	RCP experience
Patients with positive results for specific biomarkers GFAP and UCH-L1	70.00	RCP experience
Patients with negative results for specific biomarkers GFAP and UCH-L1 readmitted to the ED	0.32	RCP experience
Patients with positive results for specific biomarkers GFAP and UCH-L1 and pathological head CT	13.00	RCP experience
Patients referred for surgery after positive CT and positive result for specific biomarkers GFAP and UCH-L1	11.00	RCP experience
Patients admitted to the ICU after positive CT and positive result for specific biomarkers GFAP and UCH-L1	1.00	RCP experience
Patients in observation after positive CT and positive result for specific biomarkers GFAP and UCH-L1 who undergo follow-up CT	88.00	- **
Patients with abnormalities on follow-up CT, after positive CT and positive result for specific biomarkers GFAP and UCH-L1	100.00	RCP experience
Patients admitted to Neurosurgery with abnormalities on follow-up CT, after positive CT and positive result for specific biomarkers GFAP and UCH-L1	10.00	RCP experience
Patients referred for outpatient monitoring with abnormalities on follow-up CT after positive CT and positive result for specific biomarkers GFAP and UCH-L1	90.00	RCP experience
Patients readmitted to the ED after positive result for specific biomarkers GFAP and UCH-L1, positive CT, and negative follow-up CT ***	0.01	Ruan et al. (2009) [15]
Patients with positive results for specific biomarkers GFAP and UCH-L1 with symptoms/decrease in GCS after observation period and negative CT	5.00	RCP experience
Patients readmitted to ED due to TBI after negative CT and positive result for specific biomarkers GFAP and UCH-L1 ***	1.26	Ruan et al. (2009) [15]
Patients referred for surgery after negative CT and positive result for specific biomarkers GFAP and UCH-L1 with symptoms/decrease in GCS after observation period ***	0.123	Ruan et al. (2009) [15]
Patients with mild TBI admitted to ICU after negative CT and positive result for specific biomarkers GFAP and UCH-L1 but with symptoms/decrease in GCS after the observation period	2.00	RCP experience
Patients with symptoms/GCS < 15 undergoing follow-up CT after negative CT and positive result for specific biomarkers GFAP and UCH-L1	97.88	- **
Patients with abnormalities on follow-up CT after negative CT and positive result for specific biomarkers GFAP and UCH-L1	5.00	RCP experience
Patients admitted to Neurosurgery with abnormalities on follow-up CT after negative CT and positive result for specific biomarkers GFAP and UCH-L1	20.00	RCP experience
Patients referred for outpatient monitoring with abnormalities on follow-up CT after negative CT and positive result for specific biomarkers GFAP and UCH-L1	80.00	RCP experience
Patients readmitted to the ED after negative follow-up CT, and negative CT, with positive result for specific biomarkers GFAP and UCH-L1 ***	0.005	Ruan et al. (2009) [15]

Abbreviations: CT, computed tomography; ED, emergency department; GCS, Glasgow Coma Scale; GFAP, glial fibrillary acid protein; ICU, intensive care unit; RCP, routine clinical practice; TBI, traumatic brain injury; UCH-L1, ubiquitin C-terminal hydrolase L1. * GCS 15 with no symptoms and/or risk factors. ** The value of the third branch has been calculated as the difference between 100 and the sum of the 2 previous known values. *** For these variables, the data are based on the model published by Ruan et al. (2009) [15], which uses S100B. In this analysis, this information has been extrapolated for application to GFAP and UCH-L1. It is acknowledged that these biomarkers have differences in specificity and kinetics, which could influence the results.

**Table 2:** Costs of procedures included in the BIA.

Costs	Value (EUR)
Visit for outpatient monitoring	167
Hospital stay: admission to Neurosurgery	1240
Admission to ICU	857
Neurosurgical intervention (operating room)	1740
Repeat visit to ED	215
Observation in ED	410
Specific biomarkers GFAP and UCH-L1	32
CT	184
Standard visit to ED	215

Abbreviations: CT, computed tomography; ED, emergency department; GFAP, glial fibrillary acid protein; ICU, intensive care unit; UCH-L1, ubiquitin C-terminal hydrolase L1. NB: For procedures such as hospital stay and ICU admission, average costs of a complete admission episode were used. These estimates are based on Diagnosis-Related Group (DRG) codes most representative of mild TBI in the Spanish hospital setting, combined with averaged figures from regional tariffs. Source: data from the autonomous community tariffs and Minimum Basic Data Set (MBDS).

**Table 3:** Estimated time for each event included in the model.

Description of the Event	Min
Standard ED visit time (patients who do not require CT)	98
Time for request, preparation, transport, examination, and interpretation of the CT	156
Mean time of observation in the emergency department for patients requiring observation	319
Mean response time of the laboratory performing the test	43

Abbreviations: CT, computed tomography; ED, emergency department.

**Table 4:** Mean cost for each procedure included in the model; routine clinical practice vs. specific biomarkers GFAP and UCH-L1.

Procedure	Routine Clinical Practice	Specific Biomarkers	Difference
Emergency visits	EUR 215.00	EUR 215.00	−EUR
Specific biomarkers GFAP and UCH-L1	−EUR	EUR 28.10	EUR 28.10
CT	EUR 171.12	EUR 122.35	−EUR 48.77
Follow-up CT	EUR 48.46	EUR 20.29	−EUR 28.18
Observation in ED	EUR 376.34	EUR 268.82	−EUR 107.52
Admission to Neurosurgery	EUR 28.90	EUR 10.54	−EUR 18.36
Outpatient monitoring	EUR 34.75	EUR 12.55	−EUR 22.20
Operating room	EUR 16.90	EUR 16.29	−EUR 0.61
ICU	EUR 2.66	EUR 1.28	−EUR 1.38
Readmission to ED	EUR 4.63	EUR 4.52	−EUR 0.12

Abbreviations: CT, computed tomography; ED, emergency department; GFAP, glial fibrillary acid protein; ICU, intensive care unit; UCH-L1, ubiquitin C-terminal hydrolase L1. NB: The expected cost presented in Table 4 is calculated by multiplying the cost of each event by the cumulative probability of all decision tree branches that include that event. This cumulative probability accounts for all possible pathways where the event occurs, not just the direct probability of the event alone. This approach offers a balanced overview of the real economic impact of each event since some events that might be highly expensive have a low probability of occurrence.

## Data Availability

The original contributions presented in this study are included in the article material. Further inquiries can be directed to the corresponding author(s).

## Abbreviations

The following abbreviations are used in this manuscript:

GFAP	Glial fibrillary acid protein
UCH-L1	Ubiquitin C-terminal hydrolase L1
TBI	Mild traumatic brain injury
LD	Linear dichroism
BIA	Budget impact analysis
CT	Computed tomography
GCS	Glasgow Coma Scale
AII	Acute intracranial injury
ED	Emergency departments
SEMES	Spanish Society of Emergency Medicine
ICU	Intensive care unit
RCP	Routine clinical practice
MBDS	Minimum Basic Data Set
RECH	Spanish Network of Hospital Costs
DRG	Diagnosis-Related Group

## References

[1] Rubiano A.M., Rosenfeld J.V., Adeleye A.O., Park K.B., Shrime M.G., Hung Y.-C., Rattani A., Punchak M., Gupta S., Dewan M.C. (2018). Estimating the global incidence of traumatic brain injury. J. Neurosurg..

[2] Blennow K., Brody D.L., Kochanek P.M., Levin H., McKee A., Ribbers G.M., Yaffe K., Zetterberg H. (2016). Traumatic brain injuries. Nat. Rev. Dis. Primers.

[3] Maas A.I.R., Menon D.K., Adelson P.D., Andelic N., Bell M.J., Belli A., Bragge P., Brazinova A., Büki A., Chesnut R.M. (2017). Traumatic brain injury: Integrated approaches to improve prevention, clinical care, and research. Lancet Neurol..

[4] Freire-Aragón M.D., Rodríguez-Rodríguez A., José Egea-Guerrero J. (2017). Update in mild traumatic brain injury. Med. Clin..

[5] Faisal M., Vedin T., Edelhamre M., Forberg J.L. (2023). Diagnostic performance of biomarker S100B and guideline adherence in routine care of mild head trauma. Scand. J. Trauma Resusc. Emerg. Med..

[6] Su Y.S., Schuster J.M., Smith D.H., Stein S.C. (2019). Cost-Effectiveness of Biomarker Screening for Traumatic Brain Injury. J. Neurotrauma.

[7] Ruiz F.T., Torrecilla F.M., Sánchez M.Á.A., Gómez I.A., Bártulos A.V., España F.J.G., Román M.M., Rodríguez A.M., Morell-García D., de las Heras I.P. (2024). Traumatismo craneoencefálico leve y biomarcadores de lesión cerebral aguda. Rev. Esp. Urg. Emerg..

[8] Boice J.D., Novelline R.A., Larson P.A., Mahesh M., Linton O.W., Tenforde T.S., Bushberg J.T., Amis E.S., Timins J.K., Sierzenski P.R. (2014). Applications of justification and optimization in medical imaging: Examples of clinical guidance for computed tomography use in emergency medicine. Ann. Emerg. Med..

[9] Hoffman J.R., Nagaraj G., Sharp A.L., Swap C.J., McCormick T., Shen E., Silver M.A., Rippberger E.J., Vinson D.R. (2017). Computed Tomography Use for Adults With Head Injury: Describing Likely Avoidable Emergency Department Imaging Based on the Canadian CT Head Rule. Acad. Emerg. Med..

[10] Datwyler S.A., Bazarian J.J., McQuiston B., Jeffrey C.A., Caudle K., Chen J.Y., Chandran R., Zhang H., McCaw T., Welch R.D. (2021). Accuracy of a rapid glial fibrillary acidic protein/ubiquitin carboxyl-terminal hydrolase L1 test for the prediction of intracranial injuries on head computed tomography after mild traumatic brain injury. Acad. Emerg. Med..

[11] Augustovski F., Shau W.-Y., Lee K.M., Sullivan S.D., Orlewska E., Minchin M., Penna P., Caro J.J., Mauskopf J.A., Barrios J.-M.R. (2014). Budget impact analysis-Principles of good practice: Report of the ISPOR 2012 budget impact analysis good practice II task force. Value Health.

[12] Carswell C., Drummond M., Petrou S., Greenberg D., Augustovski F., Loder E., Briggs A.H., Mauskopf J., Pwu R.-F., Chaiyakunapruk N. (2022). Consolidated Health Economic Evaluation Reporting Standards (CHEERS) 2022 Explanation and Elaboration: A Report of the ISPOR CHEERS II Good Practices Task Force. Value Health.

[13] Bastida J.L., Oliva J., Antoñanzas F., García-Altés A., Gisbert R., Mar J., Puig-Junoy J. (2010). Propuesta de guía para la evaluación económica aplicada a las tecnologías sanitarias. Gac. Sanit..

[14] Lindner T., Vossough A., Ornato J., O Okonkwo D., Bazarian J.J., Barzo P., Peacock W.F., A Leidel B., Ludington E., Jagoda A.S. (2018). Serum GFAP and UCH-L1 for prediction of absence of intracranial injuries on head CT (ALERT-TBI): A multicentre observational study. Lancet Neurol..

[15] Ruan S., Noyes K., Bazarian J.J. (2009). The Economic Impact of S-100B as a Pre-Head CT Screening Test on Emergency Department Management of Adult Patients with Mild Traumatic Brain Injury. J. Neurotrauma.

[16] Ministerio de Sanidad Registro de Actividad de Atención Especializada. RAE-CMBD. https://www.sanidad.gob.es/estadEstudios/estadisticas/cmbdhome.htm.

[17] Grupo RECH Red Española de Costes Hospitalarios (RECH). https://www.rechosp.org/faces/es/jsf/index.jsp.

[18] Dwamena B.A., Sapin V., Oris C., Pereira B., Kahouadji S., Puravet A., Bouvier D. (2025). Can the Association of the Biomarkers GFAP and UCH-L1 Predict Intracranial Injury After Mild Traumatic Brain Injury in Adults? A Systematic Review and Meta-Analysis. Ann. Emerg. Med..

[19] Jabaudon M., Evrard B., Rouzaire P., Bourgne C., Sapin V., Rouzaire M., Duret T., Pereira B., Bouvier D., Nourrisson C. (2016). Preanalytical, analytical, gestational and pediatric aspects of the S100B immuno-assays. Clin. Chem. Lab. Med..

[20] Sapin V., Gaulmin R., Aubin R., Walrand S., Coste A., Abbot M. (2021). Blood biomarkers of mild traumatic brain injury: State of art. Neurochirurgie.

[21] Ray P., Alen J.F., Jacquin L., Durand G., Sebbane M., Maignan M., Payen J.-F., Laribi S., Morales J., Pavlov V. (2024). An automated blood test for glial fibrillary acidic protein (GFAP) and ubiquitin carboxy-terminal hydrolase L1 (UCH-L1) to predict the absence of intracranial lesions on head CT in adult patients with mild traumatic brain injury: BRAINI, a multicentre observational study in Europe. EBioMedicine.

[22] McDade C., Zimmer L., Textoris J., Krause A., Blanc E., Beyhaghi H., Pavlov V., Purser M., Earnshaw S. (2023). Cost-Effectiveness of Blood-Based Brain Biomarkers for Screening Adults with Mild Traumatic Brain Injury in the French Health Care Setting. J. Neurotrauma.

[23] Ladang A., Vavoulis G., Trifonidi I., Calluy E., Karagianni K., Mitropoulos A., Vlachos K., Cavalier E., Makris K. (2024). Increased specificity of the “GFAP/UCH-L1” mTBI rule-out test by age dependent cut-offs. Clin. Chem. Lab. Med..

